# Healthcare professionals’ experiences and attitudes to care coordination across health sectors: an interview study

**DOI:** 10.1186/s12877-022-03200-6

**Published:** 2022-06-21

**Authors:** Maiken Hjuler Persson, Jens Søndergaard, Christian Backer Mogensen, Helene Skjøt-Arkil, Pernille Tanggaard Andersen

**Affiliations:** 1 Department of Public Health, University of Southern Denmark, Degnevej 14, 6705 Esbjerg, Denmark; 2 Department of Public Health, Research Unit of General Practice, University of Southern Denmark, J. B. Winsløws Vej 9A, 5000 Odense, Denmark; 3 Emergency Department, Hospital Sønderjylland, Kresten Philipsens vej 15, 6200 Aabenraa, Denmark; 4 Department of Regional Health Research, University of Southern, Winsløwparken 19,3, 5000 Odense, Denmark

**Keywords:** Qualitative research, Individual interviews, Healthcare professionals, Care coordination, Older people, Work culture and practice, Intersectoral, Interprofessional

## Abstract

**Background:**

The number of older people is increasing, resulting in more people endure chronic diseases, multimorbidities and complex care needs. Insufficient care coordination across healthcare sectors has negative consequences for health outcomes, costs and patient evaluation. Despite introducing initiatives to solve coordination challenges within healthcare, the need remains for more consistent solutions. In particular, improved care coordination would benefit older adults characterised by complex care needs, high use of healthcare resources and multiple care providers.

**Aims and objectives:**

To identify and analyse healthcare professionals’ perspectives and approaches to care coordination across sectors when older people are acutely hospitalised.

**Design:**

Qualitative interview study.

**Methods:**

Semi-structured, individual interviews with 13 healthcare professionals across health sectors and professions were conducted. The strategy for the qualitative analysis was inspired by Kirsti Malterud and labelled ‘systematic text condensation’. This strategy is a descriptive and explorative method for thematic cross-case analysis of qualitative data.

**Results:**

Four themes/categories emerged from the analysis; “Organisational factors”, “Approaches to care”, “Communication and knowledge”, and “Relations”.

**Conclusion:**

Different organisational cultures can discourage intersectoral care coordination. Approaches to care vary at all levels across health sectors and professions. Organisational, leadership and professional identity affect the working cultures and must be considered in the future recruitment and socialisation of healthcare staff. Our research suggests that combinations of healthcare standardisations and flexible, adaptive solutions are required to improve intersectoral care coordination.

**Supplementary Information:**

The online version contains supplementary material available at 10.1186/s12877-022-03200-6.

## Introduction

The efficiency and quality of coordination of care across the healthcare system is a global challenge [1, 2], especially for older people with complex care needs [3]. Previously, concepts from social capital and relational coordination have been described as critical factors in the quality of care [4–6].

The healthcare system can be considered a ‘complex adaptive system’ (CAS) [7, 8], characterised as an open system consisting of many cyclic interacting activities on multi-dimensional levels. These elements are mutually interconnected, and the processes are dynamic and unpredictable [8, 9]. Different interactions, procedures, and practices within the system are not linear and are rarely entirely rational [8]. This complexity results from organisational requirements and structures, specialised competencies and skills, patient needs and requirements due to multimorbidity, and demographic factors [2, 10].

### Organisation of healthcare in Denmark

Denmark provides universal healthcare financed through a tax system [11]. The country has 5.8 million inhabitants, all with free access to a general practitioner (GP), gatekeepers of the Danish healthcare system [11]. GPs work within the primary healthcare sector as private practitioners but are publicly funded [11, 12].

In Denmark, healthcare and social services are divided into three administration levels; the State, five regions and 98 municipalities [13]. The State is responsible for the overall structure of healthcare, and the regions are responsible for hospital- and specialist services provided by self-employed specialists such as GPs. The 98 municipalities are responsible for health prevention and promotion at the primary level, including rehabilitation and home care services [14]. In addition, the municipalities provide personal assistance such as bathing or dressing through the Social Service Act [15, 16]. Any assistance requiring nursing expertise, such as wound care, is provided after a referral from the hospital, GP or municipality through the Healthcare Act [15, 17].

Comprehensive healthcare reform was introduced at the national level in 2007 [13]. The reform affected healthcare at other levels and resulted in further centralisation and decentralisation acts, contributing to a weakening of the regional healthcare level and a transfer of tasks and responsibilities to the municipalities (primary healthcare level) [13]. Healthcare Agreements, a tool introduced by the healthcare reform, describe how care should be coordinated across the regions, municipalities, and general practices [18]. These Healthcare Agreements are not legal documents but rather guidelines and recommendations for collaboration across health sectors. When an older person receiving homecare or nursing assistance is acutely hospitalised, the municipality sends an automated electronic record to the hospital with information about the person’s level of assistance and care at the primary sector level. If a hospitalisation exceeds 48 h, the hospital is obliged to report to the municipality describing the older person’s expected care, treatment, and post-hospitalisation needs. Later, a discharge report is formulated and sent to the municipality and GP. When the hospital evaluates the older person is ready for discharge, there is an expectation that the municipality is prepared for patient hand-over. If the person needs additional assistance after discharge, the level of service is adjusted in collaboration with the patient, next of kin, municipality, and the hospital staff.

## Background

Coordination of care is challenged when a patient is transferred between the home, nursing home, and hospital [19]. Strong collaboration within and across organisations is essential in achieving optimal care coordination [20], quality of care, patient satisfactory, and minimisation of costs [21, 22].

Initiatives to improve care coordination have been introduced at different levels with various stakeholders within the healthcare sector. However, many of these initiatives have not been fully effective [1, 2, 10]. Improved care coordination is not solely achieved with organisational and administrative solutions. Care coordination must be seen from a broader perspective, satisfying the paradox of a need for stability and standardised solutions [23] and the complexity and context-dependency of patient pathways [1, 2].

For patients enduring acute conditions, care coordination is vital. The emergency care setting is characterised by a quick turnover of aged patients with complex care needs, high use of healthcare resources between different sectors and multiple care providers [24].

Healthcare professionals’ knowledge and first-hand experiences are crucial to understanding the challenges in coordinating care services across sectors and professions [8]. Unfortunately, many studies within this field have focused on a single profession, health sector [2, 22, 25] or specific chronic illness [14]. This study is unique as it assesses the perspectives of care coordination from various healthcare professionals’ from different health sectors.

### Aim

This study aims to identify and analyse healthcare professionals’ perspectives and approaches to care coordination when an older person is acutely hospitalised.

## Method

### Study design

This article is based on qualitative interviews exploring healthcare professionals’ perceptions, approaches and experiences with care coordination across health sectors and professions when older people are acutely hospitalised. An abductive [26] and interpretative [27] approach was applied to the study combining hermeneutics and elements from phenomenology [28, 29]. Abduction describes the first stage of inquiry within which hypothesis are suggested [30]. [28, 29]No fixed theory was used before data analysis.

### Procedure

Between November 2017 and March 2018, we conducted 13 semi-structured, individual, face-to-face interviews with healthcare professionals. The healthcare professionals were asked questions about their understanding, delivery, and factors affecting interdisciplinary collaboration within the context of caring for acutely hospitalised older adult patients (See appendix). The same moderator (the first author, a female PhD student) performed all interviews. Additional interviews were conducted until no new information was revealed. Before conducting the interviews, the first author visited a municipal care team, a general practice, an out-of-hours healthcare service, and an emergency department for inspiration and an improved understanding of the different professions and settings.

### Participants

Participants were recruited as equally as possible across professions, health sectors, and municipalities using existing professional networks to establish contact with the participants. Participants included five municipal nurses, four private practitioners (GPs) and four hospital employees. All healthcare professionals worked full-time in one area/health sector. Eight participants were nurses, four were GPs, and one was a hospital physician. Eight of the informants were women. Healthcare experience varied from 10 months to 32 years, with an average of approximately 15 years of professional experience. All participants were unknown to the moderator before the interviews. All participants received written information distributed via e-mail about the study and purpose of the interview, and supplementary verbal information was given on the day of the interviews. Informed, written consent was obtained before each interview. Table 1 describes the participants’ characteristics.

**Table 1:** Participant characteristics

**Primary health sector** (Municipality and general practice)	Professional experience^*^ within healthcare	**Experience from current position**	**Duration, interview** (rounded minutes)
GP1	13 years	6 years	49
GP2	32 years	22 years	44
GP3	N/A	11 years	49
GP4	32 years	21 years	52
Municipal nurse 1	4 years	3 years, 6 months	49
Municipal nurse 2	15 years	5 years	62
Municipal nurse 3	13 years	1 year	40
Municipal nurse 4	13 years	4 years	38
Municipal nurse 5	15 years	3 years, 6 months	73
**Secondary health sector** (Hospital)			
Hospital nurse 1	14 years	1 year, 6 months	60
Hospital nurse 2	20 years	1 year	36
Hospital nurse 3	10 months	10 months	24
Physician 1	28 years	5 years	68

^*^ Months/years after basic education

### Data collection

We designed the interview guide with inspiration from Steiner Kvale [31], an extensive literature search, and observations from the initial field visits. The interview guide included questions about demographics followed by semi-structured, thematic questions about interprofessional and intersectoral collaboration and the coordination of care. This interview guide was tested on healthcare professionals and adjusted twice before the formal data collection began.

Interviews were conducted privately at the participant’s workplace. After each interview, the moderator created field notes based on reflections and observations. Each interview lasted approximately 50 min [24–73 min]. In addition, the moderator audio-recorded and transcribed the interviews verbatim using Nvivo11 for analyses.

### Analysis

The systematic text condensation was inspired by Malterud [32]. Firstly, all authors read the transcriptions carefully to become familiar with the content and develop an overview of the critical themes and topics. Secondly, we approached the coding exploratively and stepwise using an open code strategy [33]. Thirdly, we coded the data into meaning units and sub-groups. Finally, this data was sorted into four themes/categories; “Organisational factors”, “Approaches to care”, “Communication and knowledge”, and “Relations”.

Table 2 gives an example of the coding process.

**Table 2:** Examples of data analysis

Examples of Data (empiri)	Meaning unit	Sub-group	Theme/category
**Municipal nurse 5:** *“Why is it, that everything is so segmented? Who decided that when you are ONLY in orthopaedic surgery or an orthopaedic department, that you ONLY take care of that?*	Box-thinking	Structure and box-thinking	Organisational factors
**Hospital nurse 2: ***“I think it is because we do not understand how the others work. And because primarily I only have experience from the secondary (health) sector. If I had had an experience from the primary (health) sector, I would know maybe more about how the work process was there…so it is difficult to meet and feel that we completely understand each other.”*	Experience	Insight across (sectors and professions)	Communication and knowledge
**Physician 1:** *“It is actually very dangerous if you begin to define your area of responsibility too narrowly and doctors’ time is used for defensive medicine. And that caution or wariness is like a monster that can grow bigger and bigger. You end up needing bigger and bigger safety margins all the time”*	Collaborative culture	Defensive medicine	Approaches to care
**Municipal nurse 2:** *“But if we have a general practitioner who is a bit laissez-faire… We also need a feeling of security. We need to know that we have a good prescription and action plan. If we do not have that, then it does not work.”*	Confidence	Trust	Relations

The first and last authors discussed the consistency and accuracy of all coding. Finally, all authors discussed and agreed upon the interpretation of the results. These discussions ensured a systematic and transparent approach and improved the credibility and validity of the analysis [28].

### Ethical considerations and approvals

Our research was conducted in accordance with the research code of conduct (Danish Ministry of Higher Education and Science, 2014). In accordance with the Helsinki Declaration (World Medical Association, 2018), written informed consent was obtained from every informant. The study was registered by The Danish Data Protection Agency (17/31221). Upon request, the Regional Committees on Health Research Ethics from the Region of Southern Denmark has stated that the study has no notification obligation (S-20172000–135). The Committee of Multipractice Studies in General Practice recommended that GPs participate (25–2017).

### Theoretical framework

Complex adaptive system theory (CAS) shapes our fundamental understanding of the healthcare system applied in this study. Therefore, a brief introduction to this concept is necessary. Additional and relevant theory was later applied to elaborate on these results. The analysis in this study highlights that good relations, communication, trust, and respect are essential in improving care coordination aligning with elements from relational coordination (RC) [4, 34, 35]. RC was inspired by social capital theory (SC). Both RC and SC concern social interactions and relations [6], thus relevant to this study. Pierre Bourdieu, a French sociologist and anthropologist, was preoccupied with SC and related concepts (Pierre Bourdieu’s tools) [36] relevant to analysing tendencies and patterns at a practical level [37]. Finally, a theory of working culture is applied. elements are applied eclectically to the results and presented below.

### Coordination of care in a complex adaptive system

A complex adaptive system (CAS) consists of connected and interdependent elements, described as agents or fields [9]. These connections describe the complex mesh or web between agents. Interactions across agents are referred to as ‘feedback loops’ and may cause either change or stability within the CAS but are unpredictable as every CAS is unique and context-dependent [9]. Similarly, the healthcare system consists of multiple fields (health sectors or entities) and many agents (nurses, physicians or other healthcare providers). The description of an agent could also include the primary sector or general practice. Interactions consist of activities across care coordination [8, 38].

### Relational coordination

Gittell described relational coordination as a concept drawing on underlying ideas from social capital. However, the concept is embedded in organisational- and management research distinct from the origins of social capital [6, 34]. Relational coordination is an integration of services across healthcare providers and sectors through mutual trust, respect, shared knowledge and goals, and frequent, timely, accurate, and problem-solving communication [4, 5, 34, 35]. Therefore, this theory aligns well with the results of this study. In addition, Gittell suggested that social capital mediates relational coordination and organisational efficiency and performance [5]. Therefore, relational coordination is critical in collaboration between healthcare professionals’ across health sectors to deliver high-quality care.

### Social capital and concepts of Pierre Bourdieu

Social capital has origins in social science and has attracted a multitude of scholars, including Pierre Bourdieu (1930–2002) and Marcell Mauss (1872–1920) [39]. Social capital in a community can be a resource [40], a potential [41], or a process [42]. It is relevant as it describes the broader perspectives of the care continuum and collaboration across health sectors and professions. Social capital emphasises the need for mutual trust, relationships, common goals or core tasks [39, 43].

Bourdieu describes our everyday practices, such as coordinating activities in a workplace, as the result of interactions between ‘habitus’ (generative basis of actions and practices) and level of ‘capital’ (cultural, social, symbolic and economic) and ‘field’ (social arenas and settings) [36]. These interactions shape our practical logic, and the processes are never fully conscious nor unconscious [36]. Bourdieu’s description of social arenas with different and interacting fields has similarities with CAS theory [8, 38]. When this is applied to care coordination, it describes how healthcare professionals’ decisions and responses to care coordination are affected by many factors such as surroundings and the availability of resources. All social arenas (e.g. different health sectors) consist of many fields, each with their own sets of rules, values, structures and others [36]. A field is defined by a group of individual agents who endeavour to obtain control and adapt the available level of resources and capital within the field [36]. In a healthcare setting, these agents could be primary care nurses or hospital staff whose actions are determined by their practical logic [36]. The boundaries of a field are not static but determined by these agents and the extent of shared values, interests or logics within the field [36]. Collaboration and coordination of care are affected by the different logics of healthcare professionals’ practices. Bourdieu’s explanation of ‘practical logic’ refers partly to the concept of ‘culture’ but is more dynamic than a classical understanding of ‘culture’ [44].

Albertsen et al. assessed the connections, similarities, and differences between social capital and relational coordination and reported that these concepts are closely linked and, to a vast extent, overlapping [6]. They questioned whether the concepts have a mediative effect and in which direction. They suggested it was more reasonable to consider that social capital and relational coordination mutually affect one another [6] and are therefore both equally relevant.

### Working cultures within healthcare

It is not the intention of this paper to give a thorough presentation of culture as a concept. However, culture describes the accumulative sum of habits, knowledge, values, and norms present within a group at a particular time [45]. The group may share either a common educational background or workplace. In this study, ‘culture’ is applied to care coordination approaches between working and organisational contexts [46]. Including a cultural dimension gives a different understanding of the practices of healthcare professionals’. Furthermore, different organisational and working cultures affect how healthcare professionals perceive workplace logic, which shapes everyday care coordination practice [47–49]. Therefore, the results from this study are discussed with elements from the relevant theoretical concepts described above.

## Results

Four themes/categories emerged from the analysis; “Organisational factors”, “Approaches to care”, “Communication and knowledge”, and “Relations” with related sub-groups (and meaning units). The manifest level of the analysis indicated that ‘Relations’ and ‘Communication and knowledge’ are the most common themes for healthcare professionals in the coordination of care for acutely hospitalised older people. However, organisation and structural factors impact the availability of resources, relations, communication, and approaches to care and collaboration.

### Thematic content

**Fig. 1:**
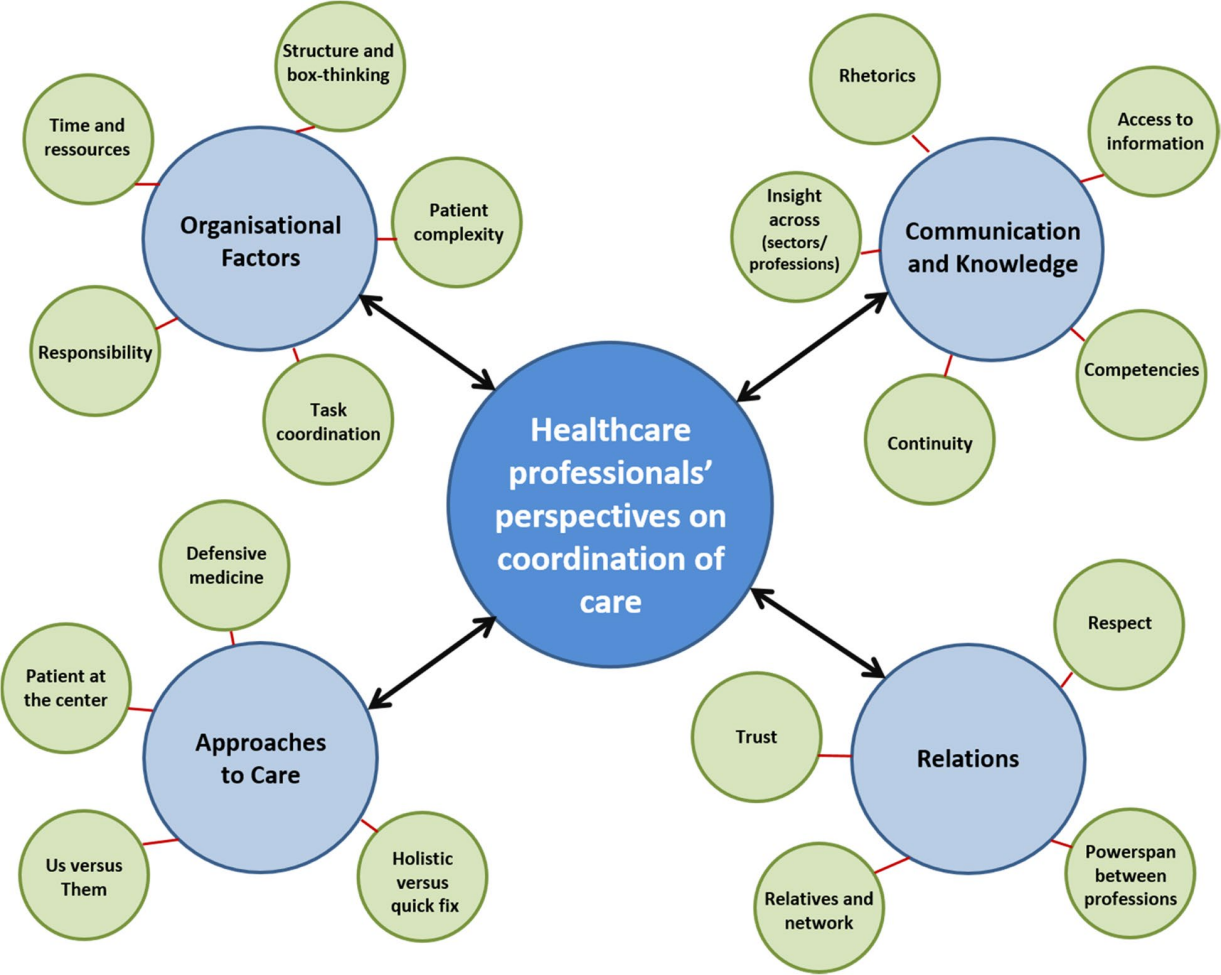
“Linking the themes and meaning units to coordination of care”. The centre, circle illustrates our research focus. The four medium-sized circles illustrate the relevant themes (categories), and the smallest are the common sub-groups identified from the analysis of the healthcare professionals’ perspectives of coordination of care. The four themes are closely interrelated in a dynamic manner

Figure 1 illustrates how the units of meaning, sub-groups and themes/categories identified in the analysis of healthcare professionals’ perspectives of care coordination across health sectors and professions are mutually interconnected, correlated and entwined in an intricate mesh pattern.

This figure only allows a two-dimensional presentation of the results. The themes/categories, in reality, are more procedurally and multi-dimensionally connected. The themes and sub-groups will be further reported below.

### Organisational factors

The healthcare system is often rhetorically referred to as one big unit or organ. In reality, healthcare is organised in and consists of multiple organisations, with individual boundaries and different structures and systems, aligning with CAS theory [9]. Over time the system has gradually become more centralised and specialised, causing demographic changes and distances between healthcare sectors and providers.

GP 3: *“There are three different systems. I think it would be different if they were all under one umbrella, but again it's about when do things become too big?. When does one large hospital become less effective compared to three small hospitals in the same city? Our organisation could maybe become too big if everything were gathered in one place. The hospital is for us a long way away. It is maybe not so much distance but the fact that we have completely lost feeling with what is happening. Of course, we get electronic letters occasionally, yeah, but it was different before.”*

In 2007, there were significant reformative changes in healthcare, and responsibility and tasks shifted between regions and municipalities, giving greater responsibility for preventive health-promoting services to the municipalities [13]. This shift caused adverse consequences for the collaboration and coordination of care.

GP 2: *”It was a considerable burden for the municipalities to take on in relation to healthcare reform….and the municipality worked subconsciously against it, not consciously of course, but they had completely different thoughts about the structure: Everyone had to have the same - in that case - worse treatment and much of the relational work was lost. It was obvious that by under prioritising the relations between health professionals from different groups, treatment of patients was worse”.*

Healthcare services and procedures are often described in detail, leaving a fragmented sense of responsibility and an ‘Everyone minds their own’ attitude to the collaboration and approaches to care. The GPs function as a gatekeeper for the patient and are often the first point of contact with the healthcare sector. However, the way healthcare is organised with rigid structures separated by sectoral borders challenges responsibility across the entire care continuum and complicates the coordination of care.

GP 2: *”It is said that General Practice is in charge of holding the string [coordinating], but that only makes sense if there is someone at the other end [of the string]. So I don’t know if I would say there is one who is responsible. There are different stakeholders. There is not just one who is responsible. It would be good to consider creating a network, so I have responsibility for my part, and I would hope that others have responsibility for theirs. But I can't take on the full responsibility for a well-connected health system, and I can’t see that others could do that either. That is something that happens in the meeting between the different healthcare professionals who are willing to collaborate and take responsibility for their part. And again, there must be good working relations to support this.“*

In Denmark, health services are controlled under two different legislations. Sometimes the language of these legislations collide.

Municipal nurse 3: *“There IS NOT a common language. What is the difference between treatment equipment and training equipment? When is something permanent? What does permanent mean? If it is not something used in the hospital, it is called treatment equipment, right? The two types of legislation clash occasionally – also in terms of the language. And when you are sitting in your own world, it can be so challenging when it is written so differently. So language, yes, but the legislation also gives challenges. I mean, these two laws, sometimes collide. Also, in relation to the terminology.”*

Therefore, the structural aspects are essential for our actions and approaches to care as they dominate how definitions are formulated. These different legislation languages create rhetoric, used as argumentation when a patient needs access to a service.

GP 1: *“It is much easier to admit (someone) to hospital than to be allocated a hospital-at-home service within a municipality – much easier. Patients with care problems [additional nursing needs] end up at the hospital … even though there is nothing medical that needs treating, it is an issue of care.”*

Similarly, economic boundaries and resources also affect the way healthcare professionals collaborate.

Municipal nurse 1: *”It can be a sick person, who, you know, their vitals are fine, but we have a feeling that there is something not quite right, and their doctor comes and has the same feeling, but because there is nothing specific we can tell the hospital, there is resistance there. Ahhh, I don’t know whether all the time in the back of our heads, that its because there are different bags of money. The economy is undoubtedly a barrier.”*

Our sense of belonging and practical logic is more influenced by our working life than a shared professional identity. Therefore, the separation between the sectors contributes to a sense of division and complicates care coordination across the health sectors.

Municipal nurse 1: *“I don't think that they (the hospital) clap their hands over their local municipality, I don’t think they do. Similarly, we are also irritated that the hospital makes mistakes again and again. I think that they stand there and say the same, that they are irritated that the municipality once again can’t do this or that. We don’t really understand each other, or we forget that everyone is pressured on all sides. It's not only oneself in our own little department.”*

Therefore, the approaches to collaboration and care are affected by both surrounding structural and organisational levels and interpersonal and individual factors such as different educational backgrounds and patient complexity. As older people are likely to experience several different illnesses, multimorbidity and complexity challenges the care coordination.

GP 1: *“If a treatment pathway becomes complex or there are no clear plans or clear distribution of responsibility, things begin to lag. If suddenly there are two diseases when a patient comes in, we can double the whole thing once more – ergo, the acute and the chronic can get in the way of each other.”*

As the GP is not directly involved in the hospital treatment and vice versa, accessing resources such as equipment from these sectors, specialties or entities complicates the overall coordination and continuum of care. Therefore, despite the need for clear descriptions, definitions and distributions of responsibilities, it is still crucial to create and find flexible solutions to accommodate patients’ individual needs and wishes.

### Approaches to care

There are differences between the primary and secondary sectors in the approach to treatment and coordination of care. These sectors operate with distinct care agendas. The practical logic of the primary sector is a holistic approach to care and treatment, whereas the hospital’s agenda is to minimise the length of admission. This dichotomy represents a challenge for coordination of care:

Physician 1: *“I think I have an important function in getting them home again quickly. I am an important function in ensuring that they are not admitted to the hospital longer than necessary. The most important from my perspective is that there is room for them that come, so we don’t have overcrowding.”*

GP 1: *“I think that we see that they come quickly in and quickly out, and so we are allowed to pick up the pieces. It is because they are busy, and there is an enormous flow of patients in and out. So there is no time to consider what I find important, its simply in and assess the patient and make a plan and move on. There is no time to wait for the background, and that, I think, really is a mistake. Maybe it can be said that the shorter the hospital stay, the better but so there ought to be another department.”*

A mismatch in expectations and logics of the patients’ best interests complicates collaboration, shared goals and care coordination for optimal treatment.

Hospital nurse 1: “*If the doctor doesn’t see the problem and just thinks, yes well a litre of saltwater and so home again…You can’t keep an admitted patient if there is no need [medical reason]. So the person comes home again, and people in the home now have a problem, and so the patient comes in again, and the primary sector gets frustrated. On the other hand, the hospital sector gets frustrated because the patient is admitted again, and the problem ought to have been solved at home. Yes, so there is very quickly dissatisfaction on both sides.”*

This frustration can cause misunderstandings and distrust between hospital staff and primary care staff. This phenomenon creates parallel working cultures and escalates a ‘them versus us’ approach, creating barriers for the coordination of care. The GP often has a thorough knowledge of the patient and their background and shares a holistic approach with primary care nurses. A shared approach (shared goals) reinforces strong alliances within a single sector across professions.

Municipal nurse 1: *“…a feeling that the GP is on the same team because we sometimes meet with them out in people’s homes and work it out together. We meet an understanding from the GP, who also is in the middle of it.*

Healthcare professionals define themselves as groups separated by sectorial borders (social arenas and fields). For example, it is a common belief among primary care nurses that hospital staff judge the primary care nurses as less competent compared to nurses employed in the hospital sector.

Municipal nurse 5: *“Sometimes I have encountered that it is ‘them and us’. Us in the municipality and them at the hospital. I have met it myself when I worked at the hospital that there is a perception that home nurses simply dish out pills and drink coffee.”*

This example demonstrates how the workplace defines ‘our team’ and ‘our logics’ more than shared professional backgrounds. However, this can differ between workplaces and organisational cultures.

Defensive medicine approaches and different roles sometimes hinder flexibility in solving tasks and coordinating care. Healthcare professionals state that focus on patient needs is lost due to the siloed and fragmented structure of the healthcare system. This suboptimal structure complicates the healthcare professionals’ ability to ensure treatment and care across sectors.

Municipal nurse 5: *“Even though we really try, the patient is outside the circle, and so it’s the practical things in the middle of the circle that are focused on rather than the patient. It’s a culture we have created so that things are task-orientated and measurable. Everything needs to be measurable today.* It *is not acceptable to provide a service that cannot be registered and measured.”*

Applying Bourdieu’s theory identifies the attempt to obtain control within a specific social arena or field. Narrow and isolated role definitions cause further fragmentation and diminish coordination of care.

GP 3: *”We know that they are busy in there [at the hospital], and all their time is used for dealing with paper and check-ups, and now they also need to - at least the younger doctors think so - need to make sure that their clinical notes are legally secured.”*

If confidence is not well-founded with each-other or the organisation, the focus on the patient can weaken, as health care professionals must act irreproachably as a defensive or protective mechanism – again, to obtain control and sense-making.

Municipal nurse 5: *”I think actually that half of my working day is spent behind a computer, and that's not why I became a nurse, because I want to be out making a difference for our citizens and that I think is damn hard because we need to be extra cautious and take preventive measures and document our way out of everything.!”*

When ‘defensive medicine’ becomes the ‘elephant in the room’, focus on tasks, time, resources, and need for documentation can overwhelm the vision of a ‘*patient-centred*’ approach.

Physician 1***:**** ”In this collaboration, you must not define your role too narrowly. It is crucial that the nurse can come with some medical ideas or reminders to the doctors. I was appalled that there are so many nurses that are just ‘ we are the carers, and they are the treaters’. That some people make such a distinction between caring and treatment. This distinction does not exist here [in the Emergency department], both parties interfere equally, and nurses are definitely part of the treatment. There are so many things here that just function automatically because people can think for themselves and take responsibility. It is actually very dangerous if you begin to define your area of responsibility too narrowly, and doctors’ time is used for defensive medicine. And that caution or wariness is like a monster that can grow bigger and bigger. You end up needing bigger and bigger safety margins all the time.”*

This statement from a physician illustrates how healthcare professionals can adapt to the complex healthcare system. However, the definition of roles is not consistent across professions:

Hospital nurse 3: *“Treatment-wise, it’s doctors (that have the responsibility). Like practically, it’s the nurses and nursing assistants in the department. If we were not with the patients, treatments would start, but there would be no-one to observe the patient – does (the treatment) work or is there something (else) that needs to be done? So Doctors are responsible for the overall treatment, and nurses do the rest.”*

These quotes confirm the likelihood of mismatched expectations of interprofessional collaboration. For example, whilst the doctor expects nurses to be flexible and adaptive in the care coordination, nurses have a more conservative expectation of their role. This mismatch may result from distinct socialisations within a workplace or different professions and educational backgrounds [46, 47].

### Relations

As reported by Gittell in the context of relational coordination [4, 34, 35], healthcare professionals consider relations, communication, and knowledge essential for optimal collaboration and care coordination. Therefore, the themes/categories ‘Relations’ and ‘Communication and knowledge’ are closely related, and reporting of these themes may overlap.

Positive experiences from earlier collaboration and familiarity facilitate better relations and a willingness to accommodate each other.

Hospital nurse 2: *”If it, in reality, should be optimised and be really good, it is necessary to see each other physically and create like social capital. Actually, having an interaction with each other has a fantastic effect. You’re MUCH more willing to help one another when you know who ‘Margaret’ is, and you’ve seen her and spoken with her and think that she’s lovely. That makes a difference the next time you are sending someone home, or you have to do something together – it’s just incredibly important to have a relationship.”*

Good collaboration with a colleague is rewarding in solving a current problem but also building trust and supporting social relations and capital. Recognising a person, a name, or a face can make collaboration more manageable, smoother, and more flexible due to familiarity. The size of a person’s team and the frequency with which the team meets impacts this sense of familiarity. Larger teams and organisations can have difficulty developing this sense of familiarity, challenging the creation of strong relations and collaborative ties.

GP 2: *“My experience has been that this was a small, well-functioning municipality, where we were close to the nurses. They came here, to the house… we knew who was good at what, and that was a major strength.”*

Good relations forge the way for building mutual trust and respect, affecting the collaboration and coordination of care. A patient’s situation is context-dependent and often unstable. The evaluation of a patient in their home environment may be entirely different from an evaluation in a hospital environment. This fact is essential when evaluating healthcare professionals’ decisions between sectors.

GP 2: *“You might also experience that if you are out visiting a very chaotic home – that you think, ‘we have to admit this person’. And then you get to the hospital, turn on the light, and there are a bunch of blood tests, the patient has been scanned, and it all seems pretty banal from a purely medical perspective.”*

Respect requires mutual understanding facilitated by insightful knowledge of each other. Misunderstandings and problematic care coordination occur with limited insight across sectoral boundaries rather than a reluctance to cooperate. According to the relational coordination theory, this could be explained by miscommunication or a lack of shared goals [4, 34]. Misunderstanding one another’s roles can inadvertently contribute to unrealistic expectations or problematic collaboration and coordination of care.

Municipal nurse 3: *”Respect for each other’s profession, respect for what we each can and can’t do. I think it's just so important. We don’t always know what the opportunities are in different sectors. We don’t always agree, that’s how it is, but respect is what ensures that we go the distance. We all need to be careful that we don’t promise something that is not doable. Our collaboration is challenged when we promise something that, in reality, you can’t promise on behalf of another sector or profession. It is often lack of knowledge or insight in how things function – it is not always easy.”*

One suggestion for improving insight into one another's roles is exchanges of healthcare staff across the sectors. For example, often, hospital employees have only had experience in a hospital setting, and vice versa for the primary care staff.

### Communication and knowledge

Precise problem-solving and positive communication to define common goals and shared responsibility supports care practice relations and coordination.

GP 2: *“It creates an enormous amount of insecurity when a patient receives completely different information…..and there must be consistent information through the whole process. It is also important that we don’t gossip about each other. And if you are uncertain, then the easiest way to assert oneself is to say that all the others are idiots!”*

This experience confirms the importance of relational coordination, demonstrating that communication and shared goals are essential for quality care and collaboration [34]. According to Gittell, the method and frequency of communication also impact care coordination [4]. Formal communication between healthcare providers is primarily electronic, and there is no common IT platform between hospital, municipalities and general practice in Denmark. This limits access and exchange of relevant information about the patient between providers.

GP 4:*”It is vital that we have some type of common platform [IT] to exchange knowledge, and we have some clear rules as to how we communicate with each other. The (correspondence module) is a good and easy way to communicate, but it also needs some rules…so I can sit at home in the evening at my home office and read when it suits me instead of me feeling that I am always pulled back and forth in the menagerie.”*

Information and knowledge are one of the cornerstones of providing quality coordination of care in a complex healthcare system.

### The dynamics of interaction and collaboration in care coordination

**Fig. 2:**
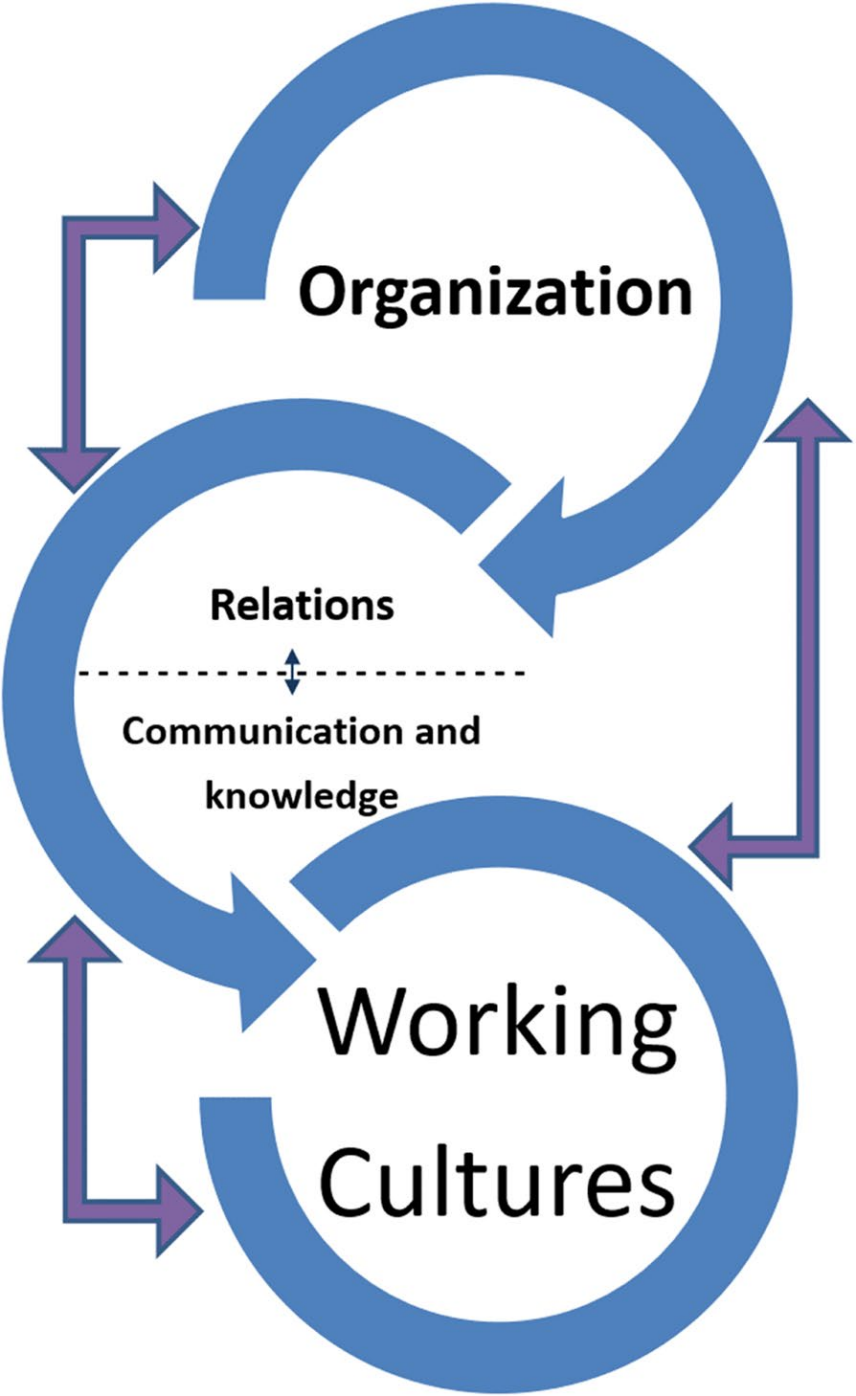
”The dynamics of collaboration and care coordination”. Figure 2 is created based on the analysis and illustrates the complex and dynamic coordination of care across and within health sectors

Figure 2 illustrates how organisation and structure, different working cultures, relations and communication between healthcare professionals and groups are mutually interconnected. It suggests how healthcare professionals interact and collaborate to coordinate care across health sectors and professions at many different levels.

Data from the interviews elaborates on this model: A GP explains that before the healthcare reform, the relationship between the GPs and municipal nurses was excellent (relations). They met physically (part of the working culture), and they knew each other well. However, after centralisation (organisational change), it was no longer possible to have these meetings (working culture changed), and their relations and familiarity with each other became weaker.

## Discussion

### Complex care coordination and working cultures

This study indicates that various levels of structure and organisation influence the coordination of care in a complex healthcare system. Similarly, a study from Sweden reported that nurses described the importance of approaching person-centred care in an acute medical ward from multiple levels, including individual, team and organisational levels [25]. Unfortunately, the Swedish study only included the perspectives of one group of professionals, the hospital nurses.

When applying Bourdieu’s theory, every sector is considered an individual field operating with different sets of norms or structures defined by physical separations, legislation, groups of healthcare professionals, structural or value-based boundaries. Healthcare professionals justify their actions and approaches to care coordination through logic sense-making. Their habits and approaches to care are partially a result of the workplace and its culture and partially shaped by professional identity, knowledge and personal experience. Furthermore, healthcare professionals’ logic sense-making and practice differ according to fields of expertise, educational backgrounds and socialisation, resulting in even more complex coordination of care. Lyngsø and colleagues assessed healthcare professionals’ perspectives on barriers and facilitators for inter-organisational care integration within the Danish healthcare system. They suggested that leaders across settings have informal meetings and healthcare staff spend time at others’ workplaces to improve coordination of care [14]. However, this study only presented a single solution to a complex problem and represented a more simplistic and linear perception of the patient pathway compared to our study.

Working cultures affect and are affected by legislation, leadership, and organisation. Further, it is recognised that healthcare professional practice and care coordination may be influenced negatively by administrative boundaries and demands [50]. Regrettably, the organisation and separation of the Danish healthcare system into primary and secondary sectors may inhibit teamwork around coordination of care and inappropriately encourages a more defensive approach to medical care and treatment, as suggested by this study.

### Relations between healthcare professionals and social capital

Improving familiarity and improving insightful knowledge and experience across health sectors, organisations, and professions would strengthen the care coordination. Leaders play a vital role in recruiting and socialising new employees to their organisation [45]. Prioritising first-hand experience by employees across health sectors is preferable, as it affects knowledge, the sense of logic and their practice. Experience across healthcare sectors builds trust, relationships, and social capital, enabling the formulation of shared goals and expectations of the core tasks in care coordination.

From the healthcare professional’s perspective, good relations with collaborative partners and first-hand insight within healthcare sectors positively affect care coordination. According to Gittel, relational coordination is a driver for coordination of care through shared goals, knowledge and mutual respect, supported by frequent, timely, accurate, problem-solving communication [4]. When the prioritisation of tasks differs between health sectors and the insight across health sectors is limited, the vision of shared goals within the health system is simply an illusion. A study from 2018 reported that patient evaluations were positively correlated with the level of organisational social capital, including shared and meaningful goals [51]. An assessment of relational coordination and social capital could contribute to knowledge about how and where to prioritise to improve care coordination.

However, relational factors are not absolute preconditions to improve care coordination as other factors such as organisational structures can either support or undermine relational coordination [4]. For example, a systematic review from 2017 reported that organisational and workplace cultures affected different patient outcomes [52]. Another study highlights that the structural level and focus on time, resources and efficiency influences how healthcare professionals approach and proceed with care for older people [12]. Thus, sector separation is a barrier to creating positive relations between healthcare providers and sectors.

### Implications for practice

Leadership and organisation should strive to recruit and socialise members to facilitate a culture of collaboration, care coordination and insight into healthcare as a complex adaptive system. Similarly, it is vital to acknowledge that education is a core institution, socialising healthcare professionals and creating a sense of professional identity. Training collaborative skills across professions could be an opportunity to improve coordination of care within the context of educational programming. In addition, further research focusing on field observations may give a more nuanced picture, as coordination of care is affected at both conscious and unconscious levels, and field observations would reveal what healthcare professionals effectuate in practice.

It is tempting to suggest that the healthcare system should not be divided into sectors. However, coordination of care is too complicated and extensive legislative reforms or restructures would simply result in a new set of challenges. Instead, the preferable solution may be to recognise the importance of competencies, qualifications and continued education. Potentially, educational institutions could improve collaboration competencies and facilitate familiarity across health sectors and professions by creating mutual knowledge and common goals. Alternatively, exchange visits by healthcare professionals across sectors may improve insightful knowledge, appreciation and engagement. In addition, exchanges would improve social relations and reduce cultural differences, benefiting coordination of care.

### Strengths and limitations

This is a strong qualitative study of intersectoral coordination of care for older people in Denmark based on individual interviews with healthcare professionals from both primary and secondary Danish healthcare sectors, including general practice. This study’s strength is that all interviews were conducted by the same person (first author), which supports the consistency of the methods [53]. Furthermore, discussing the findings among all authors with different professional backgrounds, including medicine, nursing, sociology, public health, and general practice, improves credibility [53]. This strength contributed to a more nuanced analysis of the data. Other benefits include the high degree of experience from the participants (15 years on average) and a shared background between the moderator (the first author has a healthcare professional background in midwifery) and the participants facilitating an understanding based on common language and trust. The shared background may have contributed to building trust with participants, influencing the interviews and access to knowledge positively.

We included selected groups of healthcare professionals involved in the coordination of care for older adults. Other professions, such as physiotherapists, pharmacists, or healthcare leaders, could have contributed with other insights. In addition, as the participants volunteered, recruitment may have been biased. Although these results reflect intersectoral coordination challenges in Denmark and may not apply internationally, most health systems can relate to the need for improved coordination of care.

This qualitative study contributes to understanding healthcare professionals’ perspectives on improving care coordination. However, more research is needed to assess and understand how different working cultures in a clinical setting affect care coordination.

## Conclusion

Our research presents a variety of healthcare professionals’ perspectives on care coordination across health sectors. This study highlights that working culture and cultural differences impact care coordination across health sectors. It is crucial to recognise that the healthcare system is complex and consists of independent systems with individual structures, norms and cultures, making care coordination complicated. Patient pathways are less linear and predictable than previously described, reinforcing the need for a combination of standard flexible solutions. Leadership, organisations, and educational programmes play crucial roles in the creating a culture and continuous socialisation of healthcare professionals within and across workplaces, educational institutions, health sectors and organisations.

## Supplementary Information

**Additional file 1:** Interviewguide-questions.

## Data Availability

The datasets generated and analysed during the study are not publicly available due to individual privacy given to the qualitative nature of the study. However, the data is available from the corresponding author upon reasonable request.
