# Supplementary material for: Healthcare professionals’ experiences and attitudes to care coordination across health sectors: an interview study
Source: BMC Geriatr. 2022 Jun 21;22:509. doi: 10.1186/s12877-022-03200-6 (PMC9210644; doi:10.1186/s12877-022-03200-6)
Supplement: Supplementary file 1 — Additional file 1: Interviewguide-questions. [file 12877_2022_3200_MOESM1_ESM.docx]

**Interviewguide-questions**

The following questions and discussion points were addressed after the moderator introduced herself and the project and gave initial information about the interview, such as audio recording, and expected publication of results.

**Questions for introduction**

Name and profession?

How long have you been employed in healthcare? (Municipal, regional?)

What is your current function?

How long have you had your current function?

**Issues, intersectoral and interdisciplinary cooperation:**

How do you understand the phrase *intersectoral collaboration* in relation to older persons who are acutely admitted?

How do you understand *interdisciplinary collaboration* in relation to older persons who are acutely admitted?

Who would you consider your most important partner(s) in interdisciplinary and cross-sectoral collaboration?

How do you think your partners (Hospital, Municipality, General Practitioner) see you / your professional group / your profession and function?

How do you describe your role in relation to intersectoral and interdisciplinary collaboration when an older person is acutely admitted?

In your eyes - what contributes to well-functioning collaboration across sectors and well-functioning professional boundaries?

From your perspective - what barriers can exist?

What factors are important to you to ensure cooperation before, during and after the emergency hospitalization of the older person and which factors could be strengthened / or function more optimally

**Questions, coherent patient course:**

Can you describe what you consider a "coherent healthcare patient trajectory" for an acutely admitted older person looks like?

In your eyes, what contributes to a coherent patient trajectory before, during and after emergency hospitalization of older person?

From your perspective, what barriers are there to coherence in a healthcare trajectory?

How do you regard your own role in relation to creating coherence in a patient healthcare trajectory when an older person is admitted acutely?

From your perspective, who do you consider responsible for ensuring the process is coherent?

Does a patient/person play any part in influencing the coherence of the healthcare trajectory?

Describe the role relatives have in influencing the coherence of a healthcare trajectory? (Is there a difference between relatives?) (For example, does a sparse network change your role as a health professional?)

**Concluding comments**

Is there anything relevant we have not touched on that you would like to add?

**Closing:**

- Thank you for your participation.

- How was it participating in this interview?
